# Need for Optimal Screening and Behaviour Change Interventions for Cardiometabolic Diseases in Cameroon

**DOI:** 10.7759/cureus.77784

**Published:** 2025-01-21

**Authors:** Etienne Ngeh Ngeh, Emmanuel Tito, Christopher Kuaban

**Affiliations:** 1 Physical Therapy, Sheffield Hallam University, Sheffield, GBR; 2 Medicine, Johns Hopkins University School of Medicine, Baltimore, USA; 3 Internal Medicine, University of Yaoundé I, Yaoundé, CMR

**Keywords:** cameroon, cardiometabolic diseases, disease prevention and control, lifestyle-related behaviour changes, risk factors for cardiovascular diseases

## Abstract

Cardiometabolic diseases (CMDs) are a significant public health burden in Cameroon, driven by lifestyle factors such as poor diet, physical inactivity, and tobacco use. The current response to this growing epidemic is inadequate, with limited screening and insufficient implementation of effective behaviour change interventions. To address this, a multi-pronged approach is crucial. This approach should encompass enhanced screening efforts through the integration of CMD risk factor assessments into routine healthcare visits and the implementation of community-based screening programs. Furthermore, promoting behaviour change interventions is vital, including health education, peer support groups, and training healthcare providers in motivational interviewing techniques. Strengthening healthcare systems is essential, requiring increased government investment in primary healthcare, improving access to quality care, and establishing a robust surveillance system to monitor the burden of CMDs. By implementing these strategies, Cameroon may effectively combat the rising tide of CMDs and improve the overall health and well-being of its population.

## Editorial

Cardiometabolic diseases (CMDs) are typical lifestyle-associated conditions that cannot be solely and adequately managed medically or surgically [1]. Many modifiable risk factors, such as poor diet, physical inactivity, and tobacco use, are the primary drivers of CMD incidence and prevalence across the lifespan globally [2]. Screening for these modifiable factors can lead to the adequate prevention and management of these diseases. Screening for CMDs typically involves assessing various non-communicable disease risk factors or behaviours and may be conducted individually or in combination. Multiple clustered risk factors can be assessed using tools such as the Framingham Risk Score, the World Health Organization Standardized STEPS instrument, or the Health Improvement Card, among others [3-5]. Health behaviour change interventions are safe and cost-effective approaches usually coupled with screening to tackle lifestyle conditions predisposing to CMDs. As information alone is insufficient to induce significant changes in lifestyle behaviours, health education programs are usually also designed to incorporate appropriate techniques and approaches grounded in behaviour change theories and models, which have consistently demonstrated greater success in practice [6,7]. This correspondence presents the magnitude of CMD risk factors in Cameroon that underscores the need for the country to implement robust screening and behaviour change interventions to address the situation.

Cameroon, a lower middle-income nation in Central Africa, is facing a significant rise in CMDs due to an epidemiological transition and the adoption of Westernized lifestyles [8]. The upsurge in CMDs is closely linked to the increasing prevalence of risk factors such as hypertension, diabetes, and obesity [9-13]. In 2015, a staggering 30.8% of Cameroonian women were diagnosed with hypertension, surpassing both the African average of 27% and the global female rate of 20.1% [14]. Hypertension also contributed significantly to hypertensive heart disease in the country, accounting for 41.3-54.49% of all cardiac diseases [15,16].

The prevalence of diabetes in Cameroon stands at 6% and is higher than the African average of 3.9% [17,18]. Meanwhile, a concerning 26% of the population live with hypercholesterolemia, and tobacco use among men aged 15 years and above is estimated at 43.8%, exceeding the global average of 36.1% [19-21]. Additionally, 28.5% of Cameroonian adults are physically inactive, compared to the global rate of 27.5% [19]. Meanwhile, in 2017, 37% of Cameroonian women over 25 years old were classified as overweight or obese [19]. Furthermore, it was reported in 2012 that cardiovascular diseases (CVDs) claimed the lives of 12% of the population [22]. Despite this heavy burden, a community-based study highlighted a concern for the lack of awareness of CVDs and their risk factors among the country's population [23].

Despite the strong evidence that screening is imperative for the adequate prevention and management of CMDs, recent data suggest that the conduct of CMD risk factor screening practice in Cameroon is poor and inadequate at various levels. Aminde and colleagues reported that 36%, 63%, and 45% of community dwellers were unaware of CVD risk factors, warning signs of heart attack, and stroke, respectively [23]. About 53% of Cameroonian physiotherapists reported limitations in organized practice, hindering effective screening and behaviour change interventions [24]. Ngeh and colleagues have equally reported poor screening and assessment of lifestyle risk factors in patients by allied health professionals, notably physiotherapists, in Cameroon [24,25]. The country also lacks a robust surveillance and monitoring system, with national efforts and policies primarily focused on diabetes and hypertension [26]. Meanwhile, health behaviour approaches coupled with screening are only used currently in Cameroon with a background that is focused historically on infectious diseases, notably HIV/TB and malaria [27,28]. These approaches have not been promoted among healthcare providers and public health stakeholders to address the growing epidemic of CMDs and their risk factors in the country. Broadening and integrating behaviour change intervention beyond infectious diseases would, therefore, be necessary for the fight against CMDs in the country.

To bridge the gap in the need for optimal screening and behaviour change interventions for CMDs in Cameroon, it will be essential that a paradigm shift from a "diagnose and treat" to a "predict and prevent" model be adopted. This shift should involve conducting regular screening, providing practical education, and promoting behaviour change concerning CMD risk factors. A multifaceted and collaborative approach will be required to address individual, community, and systemic barriers. Mobilizing efforts and resources and implementing evidence-based interventions across multiple levels involving all relevant sectors will be crucial. Several actions will have to be taken for its implementation to be effective.

Firstly, integrating screening and behaviour change interventions into routine primary healthcare is paramount. This necessitates training healthcare workers on screening procedures, risk assessment, and basic counseling techniques. Utilizing technology like electronic health records and mobile health technologies can improve data management and access in remote areas. Strengthening supply chains and ensuring regular equipment maintenance are crucial. Furthermore, improved coordination between different levels of the healthcare system and inter-agency collaboration are essential. Pilot programs can test different approaches and inform national strategies. This approach not only enhances early detection and management of complications but also provides a consistent framework for preventive care [29].

Secondly, community-based interventions are crucial for reaching underserved populations, particularly in rural areas. Mobile clinics and outreach programs can significantly improve access to primary prevention screening and education regarding CMDs and their risk factors. Successful mobile clinic initiatives have been documented in various African nations. For instance, in Mozambique, mobile clinics served over 23,951 individuals between October 2022 and March 2023, testing 11,782 for HIV and identifying 901 HIV-positive individuals, with 1,252 people initiating anti-retroviral therapy over the year [30]. Similarly, in Malawi, organizations like Orant Charities Africa and Wandikweza operate mobile outreach clinics to improve healthcare access in remote regions, treating over 3,000 patients monthly [31].

Thirdly, leveraging diverse communication channels is crucial. Utilizing mass media, engaging community leaders, and harnessing the power of social media platforms can effectively disseminate information about modifiable risk factors for CMDs and encourage regular screening.

Research investigating patients' perspectives on CVD health promotion in Cameroon revealed a preference for diverse delivery methods, including peer support groups. Participants emphasized the benefits of sharing experiences and challenges within such groups to improve adherence to health promotion interventions [25]. Moreover, peer-led support groups can provide invaluable social support and motivation, as demonstrated by studies in Malawi, where peer groups for diabetes management significantly improved adherence to lifestyle modifications [32,33].

Fourthly, investing in the capacity of healthcare providers is essential. Training programs in lifestyle counseling, motivational interviewing, and culturally appropriate techniques are crucial to equip healthcare providers with the necessary skills to effectively guide patients towards healthier behaviours. Ensuring that physiotherapists and nurses, who play a critical role in patient education, have access to standardized outcome measurement tools is vital for tracking progress and optimizing interventions. Easy-to-use tools that could be integrated into practice include the Motivational Interviewing Treatment Integrity (MITI) Scale, Health Improvement Card, and Diabetes Empowerment Scale (DES), among others.

Finally, sustainable funding is critical for the long-term success of health screening and education. Increased government investment in primary healthcare is essential to ensure the sustainability of screening programs and behaviour change interventions. Government subsidies to facilitate access to optimal care for those with CMD are mandatory. Other viable strategies include appropriate public-private partnerships, integration of CMD programs into broader health initiatives, and leveraging donor support effectively. Overreliance on irregular funding from donor agencies can hinder long-term program sustainability. Evidence suggests that increased government investment in primary healthcare leads to a dramatic increase in preventive care access [34,35].

In conclusion, Cameroon faces a critical challenge with the growing burden of CMDs driven by an epidemiological transition. While these strategies offer a promising approach to addressing the rising tide of CMDs in Cameroon, it is important to note that their effectiveness will depend on various factors, including their faithful implementation, adequate resource allocation, and the ongoing evaluation and adaptation of programs based on local context. Further research is needed to evaluate the effectiveness of these strategies in the Cameroonian population and to identify the most effective and cost-effective approaches.
